# Supplementary material for: Analyzing bacterial community in pit mud of Yibin Baijiu in China using high throughput sequencing
Source: PeerJ. 2020 May 12;8:e9122. doi: 10.7717/peerj.9122 (PMC7227652; doi:10.7717/peerj.9122)
Supplement: Table S1 [file peerj-08-9122-s001.docx]

**Table S1** List of pit mud samples

| **Distilleries** | **Middle** | **Sub** | **Distilleries** | **Middle** | **Sub** |
| --- | --- | --- | --- | --- | --- |
| **1** | JN-01Z1 | JN-01D1 | **10** | JN-10Z1 | JN-10D1 |
|  | JN-01Z2 | JN-01D2 |  | JN-10Z2 | JN-10D2 |
|  | —— | JN-01D3 |  | JN-10Z3 | —— |
| **2** | JN-02Z1 | —— | **11** | JN-11Z1 | JN-11D1 |
|  | JN-02Z2 | JN-02D2 |  | JN-11Z2 | JN-11D3 |
|  | JN-02Z3 | JN-02D3 |  | JN-11Z3 | —— |
| **4** | JN-04Z1 | JN-04D1 | **12** | JN-12Z1 | JN-12D1 |
|  | JN-04Z2 | JN-04D3 |  | JN-12Z2 | JN-12D2 |
|  | JN-04Z3 | —— | **15** | JN-15Z1 | JN-15D2 |
| **5** | JN-05Z1 | JN-05D1 |  | JN-15Z2 | JN-15D3 |
|  | JN-05Z2 | JN-05D2 |  | JN-15Z3 | —— |
| **6** | JN-06S2 | —— | **16** | JN-16Z1 | JN-16D1 |
|  | JN-06Z1 | —— |  | JN-16Z2 | JN-16D2 |
|  | JN-06Z2 | JN-06D2 |  | JN-16Z3 | JN-16D3 |
| **8** | JN-08Z1 | JN-08D1 |  | JN-16Z4 | JN-16D4 |
|  | JN-08Z2 | JN-08D2 | **17** | JN-17Z1 | JN-17D1 |
|  | JN-08Z3 | JN-08D3 |  | JN-17Z2 | JN-17D2 |
| **9** | JN-09Z1 | JN-09D1 |  | JN-17Z3 | JN-17D3 |
|  | JN-09Z2 | JN-09D2 |  | **Middle** | **sub** |
|  | JN-09Z3 | JN-09D3 | **Total** | **37** | **31** |
