# Supplementary material for: Analyzing bacterial community in pit mud of Yibin Baijiu in China using high throughput sequencing
Source: PeerJ. 2020 May 12;8:e9122. doi: 10.7717/peerj.9122 (PMC7227652; doi:10.7717/peerj.9122)
Supplement: Table S2 — ∗ Significant differences in a row between middle and sub layer groups as determined by ANOVA (P < 0.05) [file peerj-08-9122-s002.docx]

**Table S2** Result of physicochemical indicators of middle and sub-layer groups in PMs

| **Sampling** | **Value for indicated PM sample (mean ± SD)** | |
| --- | --- | --- |
|  | **Middle** | **Sub** |
| **moisture content (%)** | 38.03 ± 0.07 | 39.32 ± 0.08 |
| **pH*** | 6.00 ± 1.49 | 4.69 ± 1.06 |
| **ammonia nitrogen (mg/100 g)** | 58.51 ± 23.80 | 54.16 ± 22.85 |
| **available phosphorous (mg/100 g)** | 106.55 ± 118.52 | 90.31 ± 107.68 |
| **humic acid (‰)** | 3.10 ± 1.67 | 3.10 ±1.35 |

* Significant differences in a row between middle and sub layer groups as determined by ANOVA (*P*<0.05)
