# Supplementary figures and images for: Analyzing bacterial community in pit mud of Yibin Baijiu in China using high throughput sequencing
Source: PeerJ. 2020 May 12;8:e9122. doi: 10.7717/peerj.9122 (PMC7227652; doi:10.7717/peerj.9122)

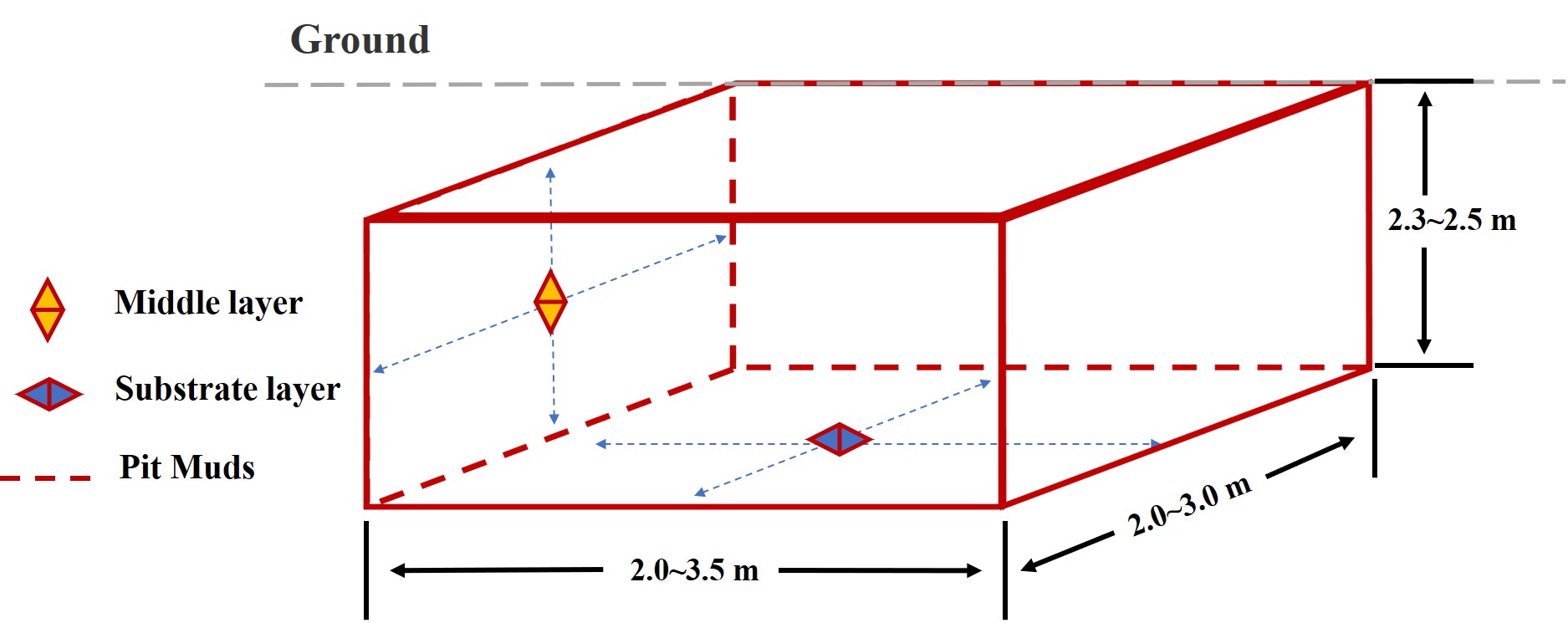

Supplement: Figure S1 [file peerj-08-9122-s003.jpg]

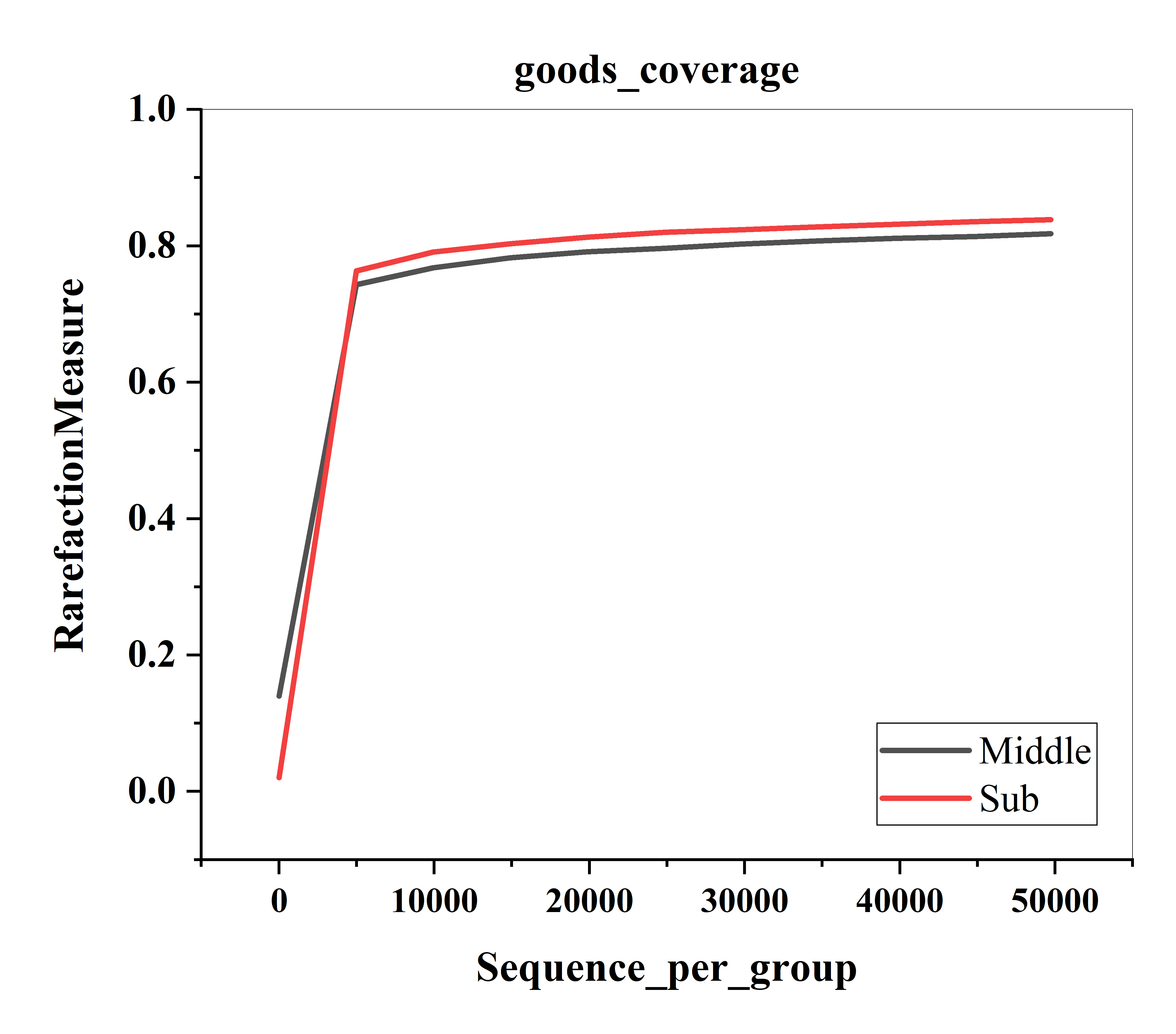

Supplement: Figure S2 [file peerj-08-9122-s004.jpg]

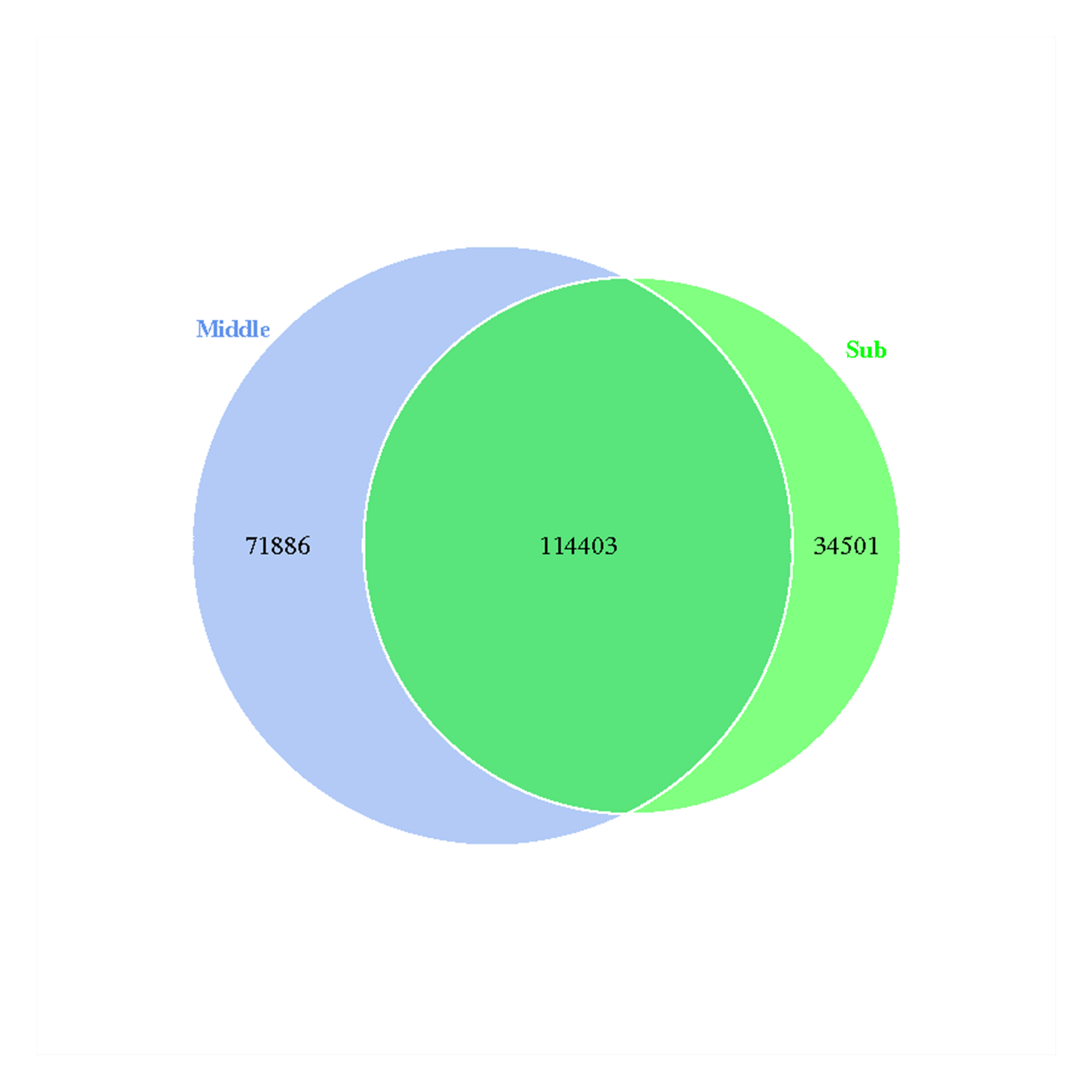

Supplement: Figure S3 [file peerj-08-9122-s005.jpg]

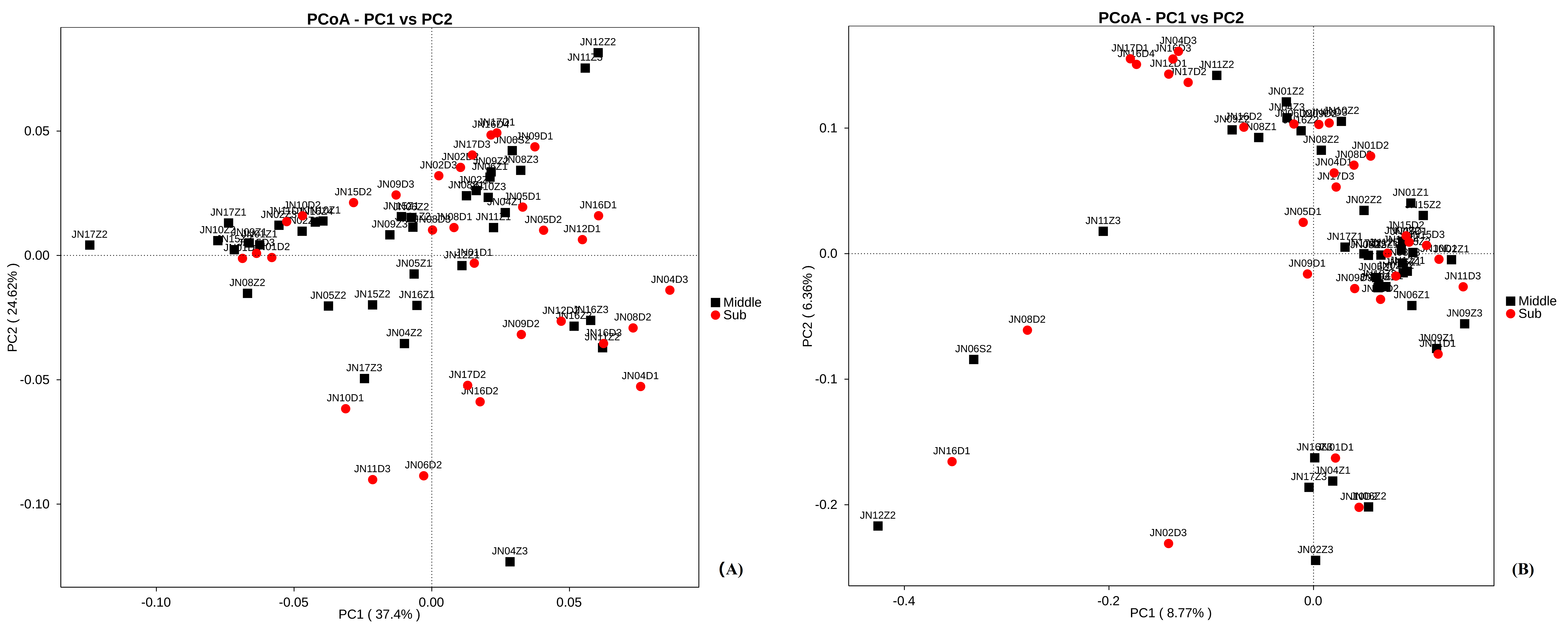

Supplement: Figure S4 [file peerj-08-9122-s006.png]

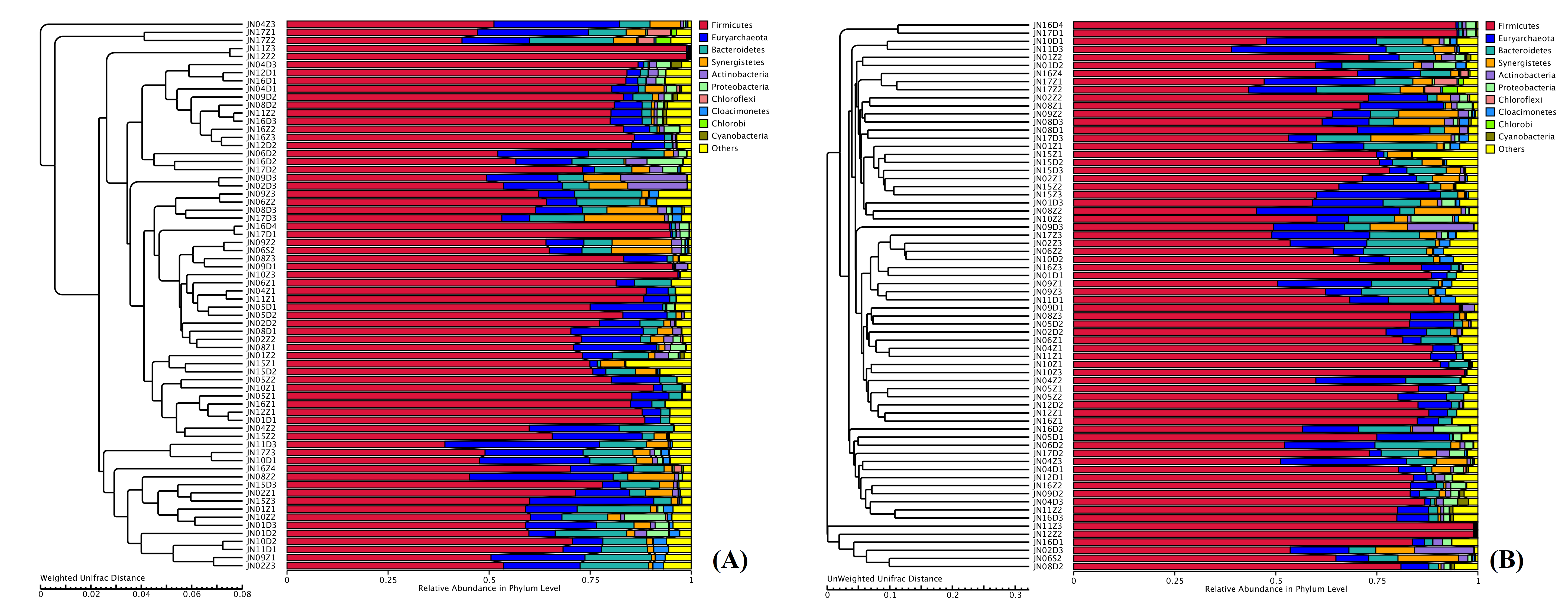

Supplement: Figure S5 [file peerj-08-9122-s007.png]
